# Hyperthyroidism complicated with diabetes mellitus using the health promotion model: changes in thyroid hormones, glucose-lipid metabolism, and inflammatory markers

**DOI:** 10.5937/jomb0-59606

**Published:** 2026-01-28

**Authors:** Miao Tang, Jiao Xu, Yanling Chen, Bo Zhou

**Affiliations:** 1 The Central Hospital of Enshi Tujia and Miao Autonomous Prefecture, Department of Endocrinology, Enshi, China; 2 The Central Hospital of Enshi Tujia and Miao Autonomous Prefecture, Department of Child Health and Rehabilitation Psychiatry, Enshi, China

**Keywords:** hyperthyroidism, diabetes mellitus, inflammatory factors, thyroid hormone, blood glucose, blood lipid, hipertireoza, dijabetes melitus, inflamatorni faktori, tiroidni hormoni, glukoza u krvi, lipidi u krvi

## Abstract

**Background:**

To investigate the effects of a Health Promotion Model (HPM)-based comprehensive intervention on thyroid hormones, glucose-lipid metabolism, and inflammatory markers in patients with hyperthyroidism (HT) complicated by diabetes mellitus (DM), and to elucidate the potential mechanisms underlying these effects.

**Methods:**

A total of 142 patients diagnosed with HT and DM between January 2024 and January 2025 were enrolled. Participants were divided into two groups: the HPM group (n = 64), which received a structured HPM-based intervention, and the conventional group (n = 78), which underwent standard management. Laboratory assessments were conducted before and after the intervention to evaluate thyroid hormones (FT3, FT4, TSH), glucose-lipid metabolism parameters (fasting plasma glucose [FPG], glycated hemoglobin [HbA1c], total cholesterol [TC], triglycerides [TG]), and inflammatory markers (high-sensitivity C-reactive protein [hs-CRP], interleukin-1p/6 [IL-1 b/IL-6]). Data were analyzed using SPSS 24.0.

**Results:**

Compared to the conventional group, the HPM group exhibited significantly greater improvements in thyroid function, with more pronounced reductions in FT3 and FT4 (P&lt; 0.001) and a greater increase in TSH (P&lt; 0.001). Regarding glucose-lipid metabolism, significantly larger decreases in FPG, HbA1c, TC, and TG were identified in the HPM group (P&lt; 0.001). Among inflammatory markers, the HPM group showed significant reductions in hs-CRP IL-1 b, IL-6, MIP-1a, and MMP-9 (P&lt; 0.001), whereas the conventional group only exhibited improvements in hs-CRP and IL-1 b (P&lt; 0.001).

**Conclusions:**

The HPM-based intervention effectively disrupts the 'thyroid-glycolipid-inflammation' axis in HT-DM comorbidity through cognitive restructuring, behavioral modification, and environmental support.

## Abstract (sr)

**Uvod:**

Cilj je ispitati efekte sveobuhvatne intervencije zasnovane na modelu promocije zdravlja (HPM) na hormone štitne žlezde, metabolizam glukoze i lipida i inflamatorne markere kod pacijenata sa hipertireozom (HT) komplikovanim dijabetes melitusom (DM), i razjasniti potencijalne mehanizme koji leže u osnovi ovih efekata.

**Metode:**

Ukupno 142 pacijenta sa dijagnozom HT i DM između januara 2024. i januara 2025. godine je uključeno u istraživanje. Učesnici su podeljeni u dve grupe: HPM grupu (n = 64), koja je primila strukturiranu HPM intervenciju, i konvencionalnu grupu (n=78), koja je podvrgnuta standardnom lečenju. Laboratorijske analize su sprovedene pre i posle intervencije radi procene tiroidnih hormona (FT3, FT4, TSH), parametara metabolizma glukoze i lipida (glukoza u plazmi na gladno [FPG], glikovani hemoglobin [HbA1c], ukupni holesterol [TC], trigliceridi [TG]) i inflamatornih markera (C-reaktivni protein visoke osetljivosti [hs-CRP], interleukin-1p/6 [IL-1|3/IL-6]). Podaci su analizirani pomoću SPSS 24.0.

**Rezultati:**

U poređenju sa konvencionalnom grupom, HPM grupa je pokazala značajno veća poboljšanja u funkciji štitne žlezde, sa izraženijim smanjenjem FT3 i FT4 (P&lt; 0,001) i većim povećanjem TSH (P&lt; 0,001). Što se tiče metabolizma glukoze i lipida, značajno veća smanjenja FPG, HbA1c, TC i TG su identifikovana u HPM grupi (P&lt; 0,001). Među inflamatornim markerima, HPM grupa je pokazala značajno smanjenje hs-CRP IL-1 b, IL-6, MIP-1a i MMP-9 (P&lt; 0,001), dok je konvencionalna grupa pokazala samo poboljšanja hs-CRP i IL-1 b (P&lt; 0,001).

**Zaključak:**

Intervencija zasnovana na HPM-u efikasno remeti osu "tiroidna žlezda-glikolipidi-upala" kod komorbiditeta HT-DM kroz kognitivno restrukturiranje, modifikaciju ponašanja i podršku iz okruženja.

## Introduction

Hyperthyroidism (HT) and diabetes mellitus (DM) are two prevalent endocrine and metabolic disorders, both demonstrating a substantial rise in global incidence in recent years [1]. Epidemiological studies reveal a striking association between these conditions, with the prevalence of DM in HT patients being 2-3-fold higher than in the general population [2]. This bidirectional relationship is further evidenced by the significantly elevated risk of thyroid dysfunction observed in individuals with DM [3]. Emerging evidence suggests a complex interplay between these two conditions at the pathophysiological level. Excessive thyroid hormones in HT exacerbate glucose metabolism dysregulation by enhancing hepatic gluconeogenesis, accelerating intestinal glucose absorption, and impairing insulin signaling [4]. Conversely, the chronic low-grade inflammation and oxidative stress characteristic of DM may potentiate thyroid dysfunction, creating a metabolic-endocrine vicious cycle [5]. This reciprocal aggravation not only complicates clinical management but also substantially increases the risk of severe complications [6]. Given these clinical challenges, elucidating the intricate relationship between HT and DM has become a priority in endocrinology research, warranting urgent investigation to improve therapeutic strategies and patient outcomes.

Although extensive research has investigated the independent pathogenesis of HT and DM, the pathophysiological mechanisms underlying their comorbidity remain poorly elucidated. Growing evidence suggests that inflammatory markers and chemokines may represent a critical pathological link between these two conditions. Specifically, abnormal thyroid hormone levels can activate the NF-kB pathway, thereby promoting the release of proinflammatory cytokines, while the resultant chronic low-grade inflammatory state exacerbates insulin resistance through interference with insulin receptor substrate phosphorylation [7]. However, a significant knowledge gap persists regarding the dynamic interrelationships among thyroid hormone levels, glycolipid metabolic parameters, and inflammatory factor networks in patients with concurrent HT and DM. Moreover, current management strategies of HT and DM are mostly limited to single disease intervention[8], and lack systematic solutions to the pathophysiological interaction between the two.

The Health Promotion Model (HPM) offers a valuable theoretical framework for addressing this clinical challenge. By emphasizing multidimensional interventions—including cognitive restructuring, behavioral modification, and environmental support—the HPM provides a novel approach to disrupting the vicious cycle of disease progression in chronic conditions [9]. The HPM's emphasis on multidimensional integration aligns with the complex pathophysiologyof endocrine-metabolic disorders, where single-disease paradigms fail to address interorgan crosstalk [10]. This pioneering study employs the HPM framework to develop comprehensive intervention strategies while simultaneously monitoring dynamic changes in thyroid function, glucose-lipid metabolism, and inflammatory biomarkers. This dual approach enables the elucidation of their complex interaction patterns, offering clinicians enhanced understanding of the synergistic mechanisms between HT and DM. Notably, this investigation represents the first integration of behavioral medicine theory with longitudinal biomarker monitoring in this context. The findings may establish a theoretical foundation for developing personalized health management protocols that transcend conventional single-drug treatment regimes. Such advancements hold substantial clinical significance for mitigating cardiovascular risk and improving long-term prognostic outcomes in this patient population.

## Materials and methods

### Study participants

A total of 142 patients with comorbid HT and DM, admitted to our hospital between January 2024 and January 2025, were enrolled in this study. The sample size was determined through a priori power analysis using G*Power v3.1 software (effect siezi = 0.3, α = 0.05, power = 0.8) and further refined based on predefined inclusion and exclusion criteria. The study protocol received approval from the Institutional Ethics Committee, and written informed consent was obtained from all participants. Among the enrolled patients, 64 were assigned to the HPM group for HPM interventions, while 78 received conventional standard care (conventional group). The baseline clinical characteristics of both groups are summarized in Table 1.

**Table 1:** Comparison of baseline data between the two groups of patients.

Groups	Age (years)	Body mass index (kg/m^2^)	Duration of HT (years)	Duration of DM (years)
Conventional (n=78)	67.59±3.24	22.89±2.58	9.35±2.41	6.95±2.81
HPM (n=64)	68.31±3.77	22.65±2.21	9.02±1.89	7.25±2.67
*t*	1.230	0.589	0.899	0.649
*P*	0.221	0.557	0.370	0.517
	Gender	Smoking	Drinking alcohol	-
	Female/Male	Yes/No	Yes/No	-
Conventional (n=78)	49/29	35/43	26/52	-
HPM (n=64)	43/21	25/39	20/44	-
χ^2^	0.294	0.486	0.070	-
*P*	0.588	0.486	0.792	-

### Inclusion and exclusion criteria

Inclusion criteria: Age ≥ 60 years; Confirmed diagnosis of both HT [11] and dM [12]; Complete clinical records; Adequate cognitive and communicative ability to participate in the study. Exclusion criteria: Acute diabetic complications; Recent major surgery or trauma (within the past month); Use of thyroid-affecting medications (within the past month); Significant organ dysfunction (e.g., hepatic, renal, or cardiac failure); Immunodeficiency disorders or active systemic infections.

### Methods

After admission, patients were treated with methimazole combined with dapagliflozin for one month, along with a 5-day therapeutic management program. In addition to the same medical treatment (methimazole + dapagliflozin), nurses provided usual management: Nurses provided oral education to patients on avoiding triggering factors such as infection and fatigue. Thyroid function and blood glucose levels were regularly monitored, and medication dosages were adjusted based on the results. Betablockers were administered during thyroid storm, while fluid replacement and insulin therapy were used for diabetic ketoacidosis. HPM: (1) Disease correlation education: Visual aids were used to explain the interaction mechanism between HT and DM to patients. 30 min each time, once a week for 4 weeks. (2) Behavioral intervention and self-management: A phased nutrition plan was developed to address the conflicting dietary demands of HT and DM (total energy 25-30 kcal/kg/d, carbohydrate proportion ≤50%, iodine intake <150 μg/d). In addition, a stepwise aerobic exercise program was designed based on thyroid function status and blood glucose fluctuations to simultaneously improve metabolic and cardiovascular health. (3) Environmental and social support: Family members received training, and a home monitoring record sheet was established to enhance dietary supervision and emotional support. Additionally, a »Thyroid-Blood Glucose Joint Screening« initiative was launched in collaboration with community health centers to provide remote dynamic monitoring and reduce follow-up dropout rates.

### Laboratory tests

Fasting venous blood samples were collected from patients upon admission and five days after management. The samples were aliquoted into coagulation-promoting tubes and anticoagulant tubes. Blood in the anticoagulant tubes was analyzed within 30 minutes of collection using a fully automated hematology analyzer (Mindray BC3000) to measure high-sensitivity C-reactive protein (hs-CRP).

Fasting plasma glucose (FPG) and hemoglobin A1c (HbA1c) levels were determined using a glucose analyzer (EKF, Biosen C-Line). For the coagulationpromoting tubes, the blood was allowed to stand at room temperature for 30-40 minutes and then centrifuged at 1500 × g for 10 minutes to separate the serum.

Total cholesterol (TC) and triglyceride (TG) levels were measured using a fully automated biochemical analyzer (Dirui CS2000). Additionally, free triiodothyronine (FT3), free thyroxine (FT4), and thyroid-stimulating hormone (TSH) were analyzed using a fully automated chemiluminescence immunoassay analyzer (Roche cobas pro e 801).

Enzyme-linked immunosorbent assay (ELISA) was performed to quantify interleukin-1β (IL-1β), interleukin-6 (IL-6), macrophage inflammatory protein-1α (MIP-1α), matrix metalloproteinase-9 (MMP-9), bone Gla protein (BGP), alkaline phosphatase (ALP), and calcitonin (CT). The assay was conducted as follows: First, standards were serially diluted using standard diluent, and serum samples (100 μL per well) were added to the plate. Each well was then washed three times with 300 μL of wash buffer, allowing 30 seconds of standing time before discarding the buffer. Next, 100 μL of biotinylated detection antibody was added to each well and incubated for one hour. Horseradish peroxidase (HRP)-conjugated streptavidin and 3, 3', 5, 5'-tetramethylbenzidine (TMB) substrate (100 μL per well) were then added for color development. The reaction was stopped by adding 50 μL of stop solution per well. Finally, the optical density (OD) at 450 nm was measured using a microplate reader (Agilent Synergy H1). All ELISA kits were purchased from Beijing Solarbio Science & Technology Co., Ltd. All assays were performed in duplicate with intra-assay CV <5%, quality control followed CLSI EP15-A3 guidelines.

### Outcome measures

The study evaluated changes in thyroid function markers (FT3, FT4, TSH), glucose-lipid metabolism parameters (FPG, HbA1c, TG, TC), inflammatory markers (hs-CRP IL-1β, IL-6, MIP-1α, MMP-9), and bone metabolism parameters (BGP ALP CT) before and after intervention. Intergroup differences were also analyzed.

### Statistical analysis

All statistical analyses were conducted using SPSS 24.0 (IBM Corp.). Categorical variables are presented as frequencies and percentages [n (%)] and were compared using chi-square tests. Normally distributed continuous variables are expressed as (χ̄±s) and were analyzed using independent samples t-tests (for between-group comparisons) and paired t-tests (for within-group comparisons). Non-normally distributed continuous variables are presented as median (interquartile range) and were compared using Mann-Whitney U tests (for between-group comparisons) and Wilcoxon signed-rank tests (for within-group comparisons). For multiple comparisons involving inflammatory markers, Bonferroni correction was applied (*P*<0.01 considered significant). A two-tailed *P*-value<0.05 was considered statistically significant.

## Results

### Changes in thyroid hormone levels

**Figure 1:**
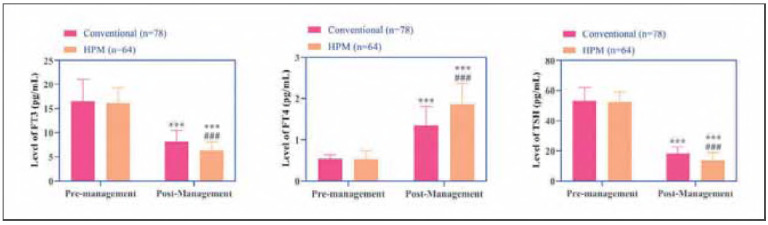
Changes in thyroid hormones FT3, FT4, and TSH before and after management in both groups.<br>Note: *** indicates the difference P < 0.001 for the intra-group comparison, and ### indicates the difference P < 0.001 for the intergroup comparison.

Both groups exhibited significant reductions in FT3 and FT4 levels alongside elevated TSH levels post-intervention (*P*<0.05), indicating enhanced thyroid function. Notably, the HPM group demonstrated more pronounced decreases in FT3 and FT4, as well as a greater increase in TSH, compared to the conventional group (*P*<0.001, Figure 1).

### Changes in glucose-lipid metabolism

**Figure 2:**
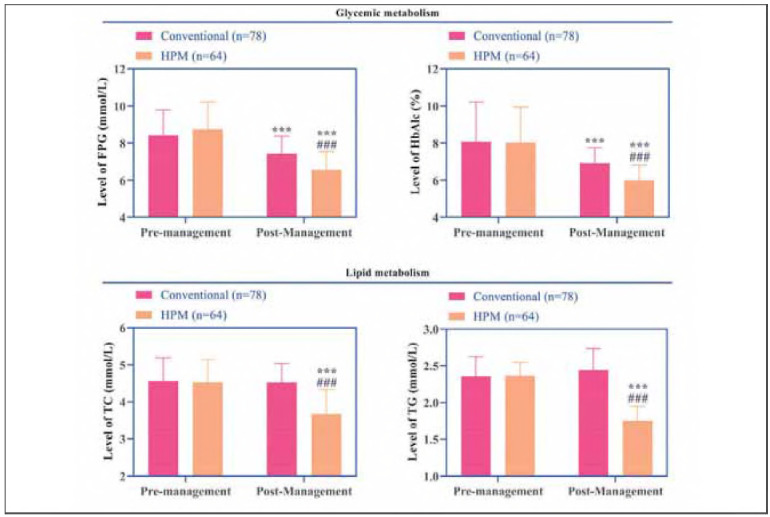
Changes in glucose (FPG and HbA1c) -lipid (TC and TG) metabolism before and after management in both groups.<br>Note: *** indicates the difference P < 0.001 for the intra-group comparison, and ### indicates the difference P < 0.001 for the intergroup comparison.

Post-intervention assessments revealed declines in FPG and HbA1c levels in both groups, with the HPM group achieving significantly lower values than the conventional group (*P*<0.001). Furthermore, while TC and TG levels remained unchanged in the conventional group, the HPM group exhibited additional reductions in these parameters (*P*<0.001, Figure 2), suggesting superior efficacy in modulating glucose-lipid metabolism.

### Changes in inflammatory markers

**Figure 3:**
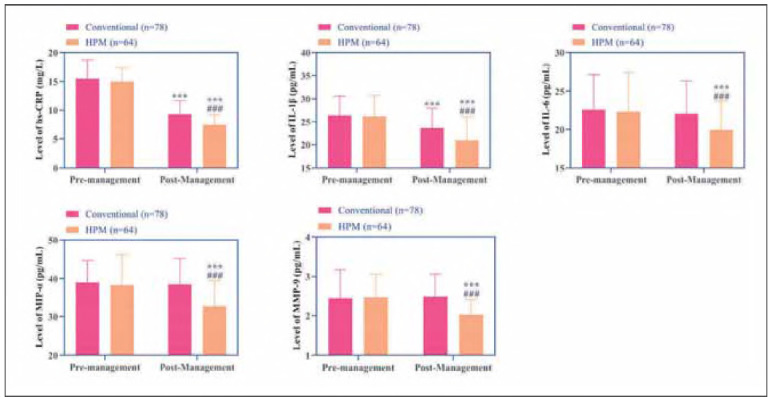
Changes in serum inflammatory factors hs-CRP, IL-1β, IL-6, MIP-α, and MMP before and after management in the two groups.<br>Note: *** indicates the difference P < 0.001 for the intra-group comparison, and ### indicates the difference P < 0.001 for the intergroupcomparison.

The conventional group showed reduced levels of hs-CRP and IL-1β post-intervention (*P*<0.001), but no significant changes were observed in IL-6, MIP-1α, or MMP-9 (*P*>0.05). In contrast, the HPM group displayed marked reductions in all measured inflammatory markers—hs-CRP IL-1β, IL-6, MIP-1α, and MMP-9—with levels significantly lower than those in the conventional group (*P*<0.001, Figure 3). These results highlight HPM's more robust antiinflammatory effects.

### Changes in bone metabolism parameters

**Figure 4:**
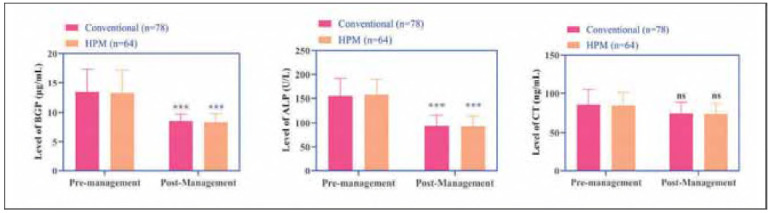
Changes in serum bone metabolic markers BGP ALP and CT before and after management in the two groups.<br>Note: *** indicates the difference P < 0.001 for the intra-group comparison.

Both groups exhibited decreased levels of BGP ALP, and CT following the intervention (*P*<0.001). However, no statistically significant differences were observed between the two groups for any of these markers (*P*>0.05, Figure 4).

## Discussion

Based on the HPM framework, this study investigated the effects of a comprehensive intervention on thyroid hormones, glucose-lipid metabolism, and inflammatory markers in patients with HT comorbid with DM. The results demonstrated that the HPM group exhibited significantly greater improvements in thyroid function, glucose-lipid metabolism, and inflammatory response compared to the conventional group. However, no significant intergroup differences were observed in bone metabolism parameters [12].

In the HPM group, serum levels of FT3 and FT4 decreased significantly, while TSH levels showed a more pronounced increase. These findings suggest that the HPM-based intervention may optimize thyroid function regulation through multiple mechanisms. First, the »disease correlation education« component enhanced patients' understanding of the pathophysiological interplay between HT and DM, thereby improving treatment adherence and contributing to better control of thyroid hormone secretion. Second, the »phased nutrition plan« likely mitigated thyroid stimulation by reducing high-iodine and high-glycemic dietary intake, while the »stepwise aerobic exercise« component may have indirectly suppressed excessive thyroid hormone synthesis by modulatingsympathetic nervous system activity [13] [14]. Furthermore, the integration of home monitoring andremote dynamic management reduced patient dropout rates and ensured timely medication adjustments. These observations align with previous studies reporting that behavioral and lifestyle interventions can significantly improve treatment adherence in DM patients [15]. Additionally, the HPM group demonstrated more pronounced reductions in FPG, HbA1c, TC, and TG levels, suggesting a synergistic effect of HPM in improving glucose-lipid metabolism. Mechanistic analysis reveals that behavioral modifications—including dietary control and structured exercise regimens—likely enhance insulin sensitivity by reducing visceral fat accumulation, thereby optimizing glucose metabolism [16]. Furthermore, environmental support mechanisms (e.g., family supervision) contribute to reduced consumption of high-fat diets, while remote monitoring enables dynamic adjustments of hypoglycemic medications, minimizing glycemic variability. Notably, the observed decreases in TC and TG levels in the HPM group imply potential modulation of lipid metabolism through inhibition of hepatic lipase activity or promotion of fatty acid oxidation pathways. In contrast, the conventional group, which relied exclusively on pharmacological adjustments without sustained lifestyle interventions, showed only marginal improvements in glucose-lipid metabolism parameters. Regarding inflammatory markers, the HPM group exhibited significant reductions in hs-CRP IL-1β, and IL-6 levels, at the same time, the observed reductions in MIP-1α and MMP-9 specifically implicate attenuated macrophage activation and extracellular matrix remodeling, aligning with NF-kB pathway suppression. According to behavioral medicine principles, stress reduction techniques and psychosocial support likely contributed to lowered cortisol levels, subsequently attenuating pro-inflammatory cytokine release [17] [18]. Additionally, existing research demonstrates that regular physical activity can counteract inflammatory cascades through upregulation of anti-inflammatory mediators like IL-10 [19] [20]. By comparison, the conventional group showed only partial improvement in select inflammatory markers (hs-CRP IL-1β), potentially attributable to the known anti-inflammatory properties of certain medications (e.g., dapagliflozin) [21]. The absence of comprehensive lifestyle interventions in this group may explain the lack of significant changes in other inflammatory parameters such as IL-6. At the sametime, the absence of intergroup differences in bone metabolism markers might be attributed to the short intervention duration (5 days), insufficient to alter bone remodeling dynamics.

We believe that the notable advantages of the HPM intervention model can be attributed to its comprehensive, multidimensional framework. Specifically, the incorporation of visual aids significantly enhances patients' comprehension of disease interaction mechanisms, thereby improving self-management efficacy through cognitive restructuring [22]. Furthermore, the tailored, phase-adapted nutrition and exercise protocols effectively address metabolic conflicts, preventing therapeutic contradictions between HT and DM management (behavioral modification) [23]. Moreover, the family-community support network establishes a robust, continuous monitoring system that mitigates the negative impact of external environmental factors on disease progression (environmental reinforcement). In contrast, conventional approaches relying solely on verbal education and passive medication adjustments fail to address the fundamental aspects of disease management, consequently limiting intervention efficacy. This finding aligns with the study by Fu J et al. [24], which demonstrated the superior effectiveness of multidimensional interventions in chronic disease management among hypertensive patients.

Based on these evidence-based findings, we strongly recommend the clinical implementation of the HPM framework, which integrates disease education, behavioral modification, and environmental support into standardized clinical pathways through multidisciplinary collaboration. Additionally, leveraging remote monitoring technologies to establish a »thyroid-glucose co-management« system could substantially reduce patients' follow-up burden. Importantly, nutrition and exercise regimens should be dynamically adjusted according to fluctuations in thyroid function and glycemic control to optimize healthcare quality and improve long-term patient outcomes.

Certainly, this study is not without limitations that warrant careful consideration. The sample size was small, and the acute 5-day observation window may capture immediate biochemical responses but not longer-term clinical outcomes, such as reductions in cardiovascular events. Furthermore, although the HPM theory emphasizes psychological determinants, the current investigation failed to incorporate relevant psychological assessments (e.g., anxiety or depression scales). The absence of significant differences in bone metabolism parameters between groups could potentially be attributed to either the truncated observation window or the non-specific nature of the interventions regarding bone metabolism, highlighting an important avenue for future research. In addition, considering the association between HT and DM, the sequence of occurrence of the two diseases may have an important effect on the results of the study. In follow-up studies, we also plan to prospectively include the order of disease onset as a key variable for future studies, which will allow us to rigorously test the effects of HPM on metabolic control, inflammation, and other clinical endpoints.

## Conclusion

The HPM theory offers a novel paradigm for managing concurrent HT and DM by disrupting thepathogenic »metabolism-endocrine-inflammation« triad. Subsequent studies should prioritize larger-scale investigations with extended follow-up durations to validate these preliminary findings. Additionally, more rigorous exploration of the mechanistic links between behavioral modifications and molecular pathways would significantly advance our understanding in this field.

## Dodatak

### Availability of data and materials

The data that support the findings of this study are available from the corresponding author upon reasonable request.

### Conflict of interest statement

All the authors declare that they have no conflict of interest in this work.
